# Miller Fisher Syndrome in the COVID-19 Era – A Novel Target Antigen Calls for Novel Treatment

**DOI:** 10.7759/cureus.12424

**Published:** 2021-01-01

**Authors:** Surina Kajani, Raheem Kajani, Chin-Wei Huang, Tu Tran, Antonio K Liu

**Affiliations:** 1 Medicine, Adventist Health White Memorial, Los Angeles, USA; 2 Critical Care, Los Angeles Community Hospital, Los Angeles, USA; 3 Gastroenterology, Los Angeles Community Hospital, Los Angeles, USA; 4 Neurology, Adventist Health White Memorial, Los Angeles, USA

**Keywords:** miller fisher syndrome, gq1b antibody, covid-19

## Abstract

Although Miller Fisher syndrome cases have been published in this coronavirus disease 2019 (COVID-19) pandemic, anti-GQ1b antibody has not been identified so far. A direct proof of association is not yet available since the exact pathophysiology is not known. Using a proof of contradiction argument, lack of GQ1b serves as the indirect proof that severe acute respiratory syndrome coronavirus 2 (SARS-CoV-2) is probably the infection preceding demyelination. A novel antigen has yet to be described.

## Introduction

It is well established that Guillain-Barre syndrome (GBS) can be associated with severe acute respiratory syndrome coronavirus 2 (SARS-CoV-2) infection. Miller Fisher syndrome (MFS), a variant of GBS, has also been described [1-5]. A literature review revealed at least six MFS patients (one from Italy, four from Spain, and one from New York, USA). We present a case of MFS in a patient with SARS-CoV-2 in Los Angeles in June 2020. So far, no patient has tested positive for anti-GQ1b antibody. Prior to 2020, anti-GQ1b antibody has been reported to be present in 81% of patients with MFS [6]. Assuming molecular mimicry is still the underlying mechanism, the human immune response could be targeting a different protein other than GQ1b after SARS-CoV-2 infection. The majority of MFS-associated anti-GQ1b antibodies cross-react with GT1a and other disialylated gangliosides, including GD1b and GD3 [7]. It is known that GD3 and arginylglycylaspartic acid (RGD) are involved in cell adhesion mechanism [8]. RGD mortif has been suggested as an alternate receptor for SARS-CoV-2 [9]. There is likely a new kind of autoimmune antibody formation that subsequently reacts with other known proteins resulting in MFS. Besides presenting an anti-GQ1b antibody negative MFS case, we also look into the emerging evidence that administration of calcium channel blockers may benefit coronavirus disease 2019 (COVID-19) management as an adjunctive therapy and suggest possible trials on MFS and GBS. 

## Case presentation

A 50-year-old male with a past medical history of obesity, type 2 diabetes mellitus, and heroin use presented to the emergency department (ED) with four days of slurred speech and progressive difficulty with swallowing. He denied any shortness of breath, fevers, cough, chills, or respiratory issues. On arrival to the ED, the patient had a temperature of 98.4 F, heart rate of 108/min, blood pressure of 117/78 mmHg, a respiratory rate of 19 breaths per minute, and oxygen saturation (SpO_2_) 94% on room air. On physical exam, the patient had a normal cardiac examination, and his lungs were clear to auscultation. He was oriented and coherent. It was noted that he had palate weakness, exhibiting an obvious soft nasal tone in his voice. An eye exam revealed bilateral ptosis and generalized internal and external ophthalmoplegia in all directions; there was no nystagmus. There was no fluctuation or fatigability with time. On motor examination, he had normal tone and bulk. Upper extremities strength was 5-/5 and lower extremities strength was 5/5. Reflexes in the patient’s upper extremities were absent. In the lower extremities, he showed slight patellar reflexes. He had mild dysmetria and dysdiadochokinesia in the upper extremities. Laboratory studies were significant for a positive SARS-CoV-2 polymerase chain reaction (PCR) from a nasopharyngeal swab. He had normal comprehensive chemistry, creatine kinase and complete blood count.

**Figure 1:**
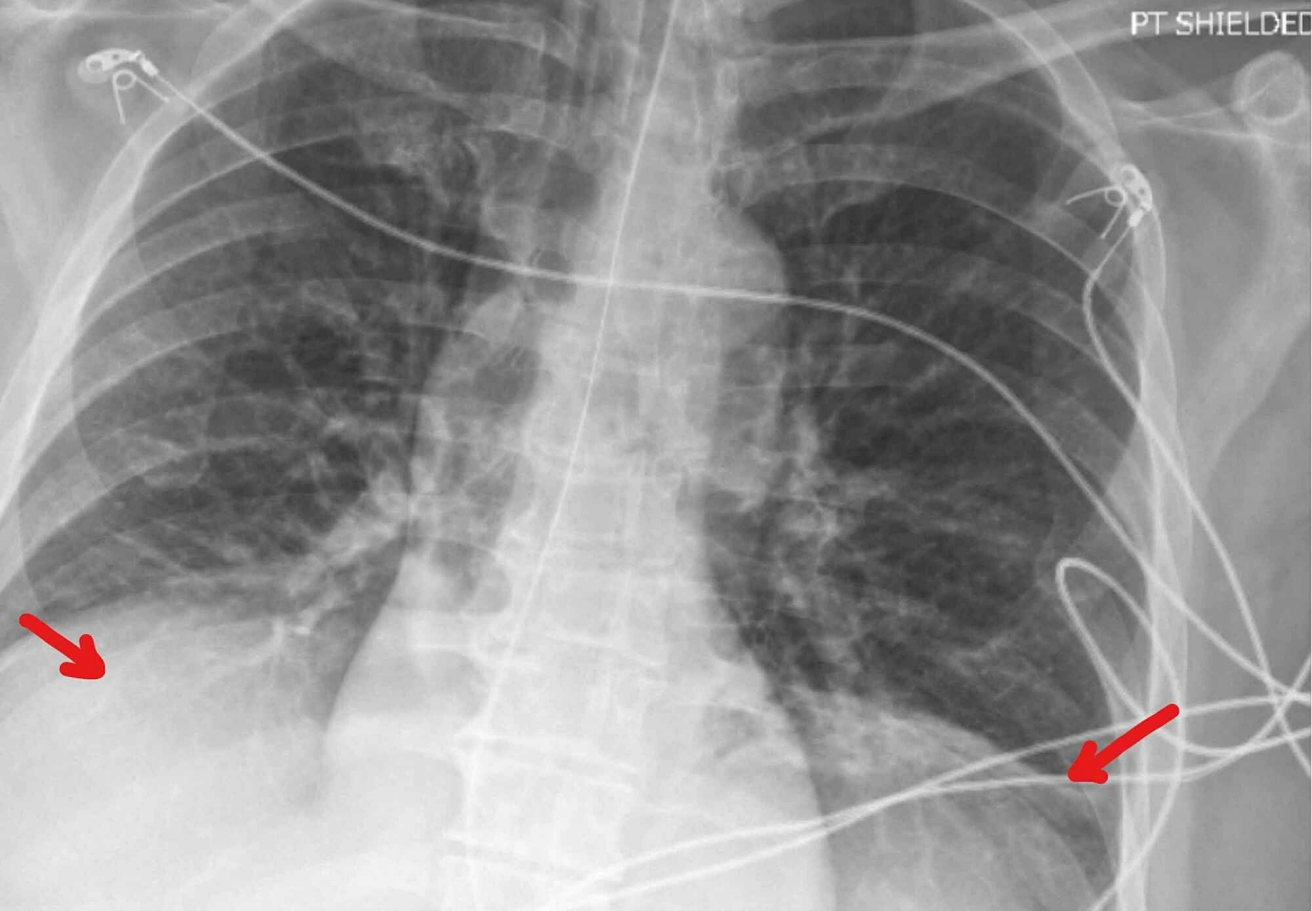
Chest radiograph showing bilateral basilar atelectasis and small pleural effusions

The patient was found to have increasing difficulty protecting his airway and was subsequently intubated. CT and MRI of the head were normal. Initial chest radiograph and chest CT were also normal. Subsequent chest radiograph only showed bilateral basilar atelectasis and small pleural effusions (Figure 1). His descending weakness progressed to his shoulder, proximal upper extremity as well as muscles of respiration. Negative inspiratory force (NIF) was persistently low. His mentation remained intact throughout his hospitalization, having to communicate via hand gestures and writing. His spinal fluid analysis showed zero white blood cells (WBC), zero red blood cells (RBC), protein of 47 mg/dL (range 15-45), and cerebrospinal fluid (CSF) SARS-CoV-2 PCR was negative. Serum antibodies (both immunoglobulin M [IgM] and immunoglobulin G [IgG]) in the ganglioside antibody panel (GM1, GD1a, GD1b, GQ1b and ASIALO GM1) were all negative. Serum acetylcholine receptor antibodies were also negative. No neurophysiological study was performed. The patient was started on IV immunoglobulin (IVIG) at 400 mg/kg/day intravenously daily for five days. On hospital day 7, approximately 12 days after symptoms onset, the patient began showing some improvements. The patient was able to open his eyelids partially, as well as partially gaze to both sides. Unfortunately, on hospital day 9, the patient developed ventricular arrhythmia and suffered a cardiac arrest unexpectedly. Despite prolonged resuscitation efforts of more than 45 minutes, the patient passed away. 

## Discussion

In our patient, despite the ganglioside antibody panel being negative, he exhibited the cardinal signs of MFS: descending weakness with ophthalmoplegia, ataxia, and areflexia. Furthermore, the patient's improvement after IVIG treatment also supported the MFS diagnosis. Of note, the patient never presented with hypoxemia or shortness of breath. Multiple imaging modalities, including CT chest and chest radiograph (CXR), did not show any evidence of any infiltrates that are typical of COVID-19 and other viral pneumonias. As aforementioned, the CSF SARS-CoV-2 PCR was negative, suggesting that the bulbar weakness experienced by the patient was not a direct effect of SARS-CoV-2 within the central nervous system. Unfortunately, we did not have neurophysiological studies to further support our diagnosis. A review of the literature showed at least six patients with MFS associated with COVID-19. Several review papers also exist, all referring to the same six cases [1-5]. It appears that all six cases were diagnosed by means of clinical presentation as well as response to treatment; neurophysiological studies were performed only on two of the six cases. Only one had a definite positive antibody (GD1b). A case of MFS affiliated with anti-GQ1b antibody during this pandemic has yet to be seen. 

The fact that the immunological profile of MFS is different from before the COVID-19 pandemic is already the in-direct proof that there is an association between MFS and COVID-19. Using the proof of contradiction argument, the statement "COVID-19 is not associated with MFS" cannot be true because the statement "anti-GQ1b antibody is not affiliated with MFS" is also untrue. We have seen surges of MFS (independent of overall GBS) before [10]. In downtown Los Angeles, a surge of MFS was observed in 2015, accounting for 50% of all GBS cases; anti-GQ1b antibody was found in 50% of the MFS patients.

Identifying the responsible target antigen and understanding how it fits into the demyelination pathophysiology is important for the development of safe and effective vaccines as well as strategies to alleviate patient symptoms. Our understanding of GBS and MFS is incomplete; anti-GQ1b antibody is just part of the clinical picture. We have yet to explain why anti-GQ1b antibody can manifest in so many different pathologic ways. Aside from MFS, anti-GQ1b antibody is also associated with Bickerstaff’s brainstem encephalitis (BBE) [11]. Only sera identified in anti-GQ1b antibody positive BBE patient have been shown to disrupt the blood brain barrier and caused brainstem pathology; the presence of anti-GQ1b antibody in itself is not the determining factor. It has been shown that SARS-CoV-2 gains entry into our body mainly via angiotensin-converting enzyme 2 (ACE 2) receptors. It is known that GD3 (which has cross-reactivity with GQ1b) is involved in cell adhesion mechanism along with RGD [8]. It has been suggested that SARS-CoV-2 may also gain entry to a host cell via the RGD motif [9]. More studies in the future are needed to explore these kinds of cross-reactivities and their roles in the pathophysiology. Since we do not know what protein structures other than GQ1b are our immune system targeting with SARS-CoV-2, how do we ensure that the new RNA vaccines are not causing susceptible individuals from developing autoimmune disease? 

From a treatment standpoint, articles suggest the possible benefit of calcium channel blocker as an adjunct therapy in COVID-19 patients [12, 13]. Calcium influx and calcium channel function may be involved in the pathophysiology of SAR-CoV-2 invasion of the host cell. Furthermore, RGD interaction is affected by calcium influx [9]. Ganglioside, including GQ1b, has a close relationship with calcium inflow also [13]. Further studies are needed to look into the effectiveness of such in the treatment of GBS and MFS.

## Conclusions

COVID-19 is probably associated with MFS. The patient in our case has a similar clinical presentation and response to treatment to MFS prior to the COVID-19 era. Since GQ1b has not been identified, a new novel antigen is probably involved as the target of our own immune system. Identifying this new novel antigen will further enrich our understanding of COVID-19, GBS and MFS. It will also help with the fight with the current pandemic from a medication development standpoint. Finally, for vaccine development, will targeting this "novel antigen" or other spike proteins lead to demyelination after vaccination is to be carefully monitored.
